# Supplementary material for: Genetic Dissection of Spike Productivity Traits in the Siberian Collection of Spring Barley
Source: Biomolecules. 2023 May 30;13(6):909. doi: 10.3390/biom13060909 (PMC10295979; doi:10.3390/biom13060909)
Supplement: Supplementary file 1 [file biomolecules-13-00909-s001.zip › Figure S1 QQplots_EMMAX.pdf]

Supplementary figures. QQ-plots of traits built in the EMMAX program.

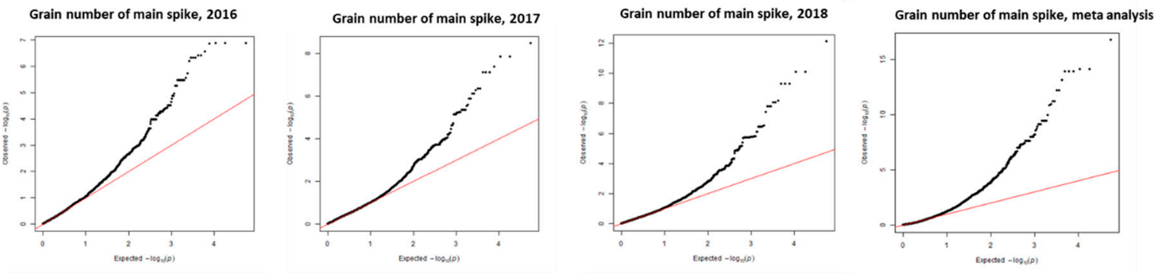

Figure S1. Grain number of main spike

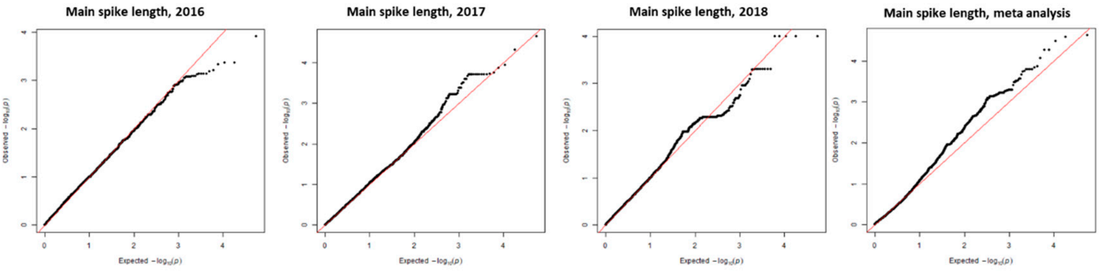

Figure S2. Main spike length

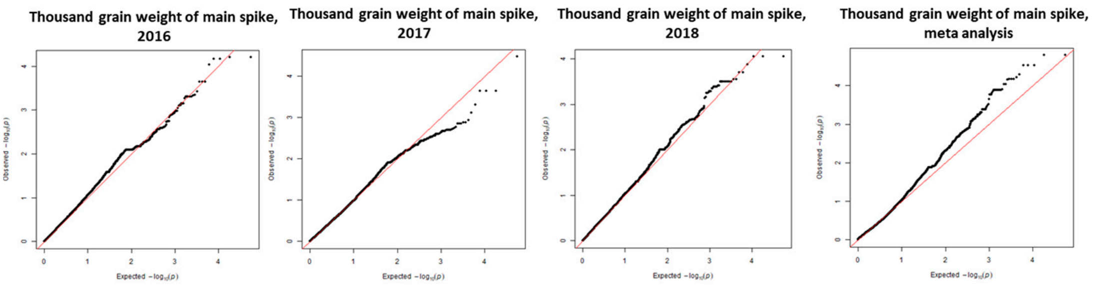

Figure S3. Thousand grain of main spike

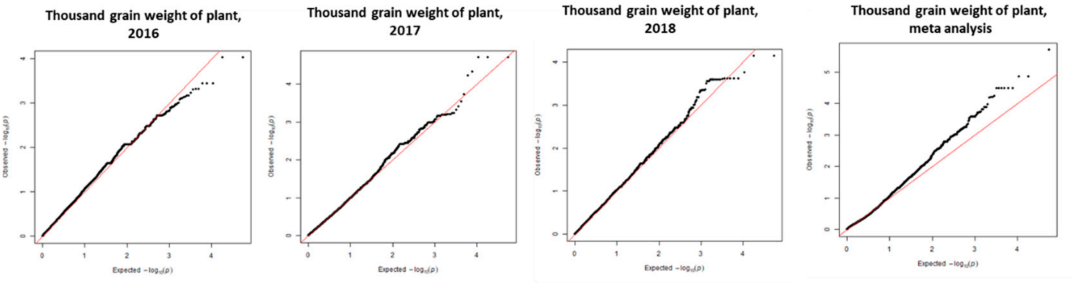

Figure S4. Thousand grain of plant
