# Supplementary material for: Genetic Dissection of Spike Productivity Traits in the Siberian Collection of Spring Barley
Source: Biomolecules. 2023 May 30;13(6):909. doi: 10.3390/biom13060909 (PMC10295979; doi:10.3390/biom13060909)
Supplement: Supplementary file 1 [file biomolecules-13-00909-s001.zip › Table S1 Siberian collection varieties..pdf]

Table S1. Siberian collection varieties.

Variety name	Row/Type	Katalog № ICG/VIR	Country, region RF
Abyssinian	nudum	ICG-8462/K-27672	Ethiopia
AC 0760258	nutans	ICG-8518	NA
Acha	nutans	ICG-8028/K-30243	Russia, Novosibirsk region
Agul	ricotense	ICG-8161/K-24707	Russia, Krasnoyarsk region
Alag-Erdene	nudum	ICG-8504/K-29415	Yemen Arab Republic
Aley	nutans	ICG-8516	Russia, Altai region
Altan-Bulag	nutans	ICG-8218/K-29268	Russia, Buryatia
Alyn-Buya	nutans	ICG-8434/-	Kazakhstan
Anna	nutans	ICG-8413/K-30829	Russia, Orenburg region
Archekas	nudum	ICG-8464/-	Russia, Kemerovo regiono
Arna	nutans	ICG-8517	Kazakhstan
Avalon	nutans	ICG-8515	France
B-1	pallidum	ICG-8436	Russia, Novosibirsk region
Bagan	nutans	ICG-8425/K-29040	Russia, Novosibirsk region
Barkhatny	ricotense	ICG-8316/K-30891	Russia, Tyumen region
Belogorsky	pallidum	ICG-8177/K-22089	Russia, Leningrad region
Bezenchuksky 2	pallidum	ICG-8267/K-30799	Russia, Samara region
Biom	nutans	ICG-8519 K-30984	Russia, Novosibirsk region
Brodyole	coeleste	ICG-8071/K-24634	USA
Chelaybinsky 70	nutans	ICG-8158/K-29470	Russia, Chelyabinsk region
Dobry	pallidum	ICG-8283/K-29215	Russia, Kirov region
Donetsky 8	medicum	ICG-8053/K-23682	Ukraine
Emelya	ricotense	ICG-8527	Russia, Krasnoyarsk region
G-19951	nutans	ICG-8522	Russia, Novosibirsk region
G-19980	nutans	ICG-8523	Russia, Novosibirsk region
G-21038	nutans	ICG-8524	Russia, Novosibirsk region
G-21219	nutans	ICG-8525	Russia, Novosibirsk region
G-21671	nutans	ICG-8526	Russia, Novosibirsk region
Golozyorny 1	nudum	ICG-8268/K-21694	Russia, Sverdlovsk region
Granal	nutans	ICG-8152/K-23342	Kazakhstan
Ilmen	nutans	ICG-8027/K-26968	Russia, Sverdlovsk region
Impuls	nutans	ICG-8260/K-29435	Russia, Sverdlovsk region
Jngve	nutans	ICG-8514/K-30023	Sweden
Kedr	pallidum	ICG-8271/K-28119	Russia, Krasnoyarsk region
Kolchan	ricotense	ICG-8435/K-31039	Russia, Altai region
Krasnoyarsky 1	nutans	ICG-8259/K-19829	Russia, Krasnoyarsk region
Krasnoyarsky 91	pallidum	ICG-8528	Russia, Krasnoyarsk region
Krymchak 55	pallidum	ICG-8127/K-25286	Ukraine
Kuryer	nutans	ICG-8246/K-26740	Russia, Krasnodar region
L-1	nudum	ICG-8456	Russia, Novosibirsk region
L-1285	nudum	ICG-8451	Russia, Novosibirsk region
L-259/528	ricotense	ICG-8044/-	Russia, Novosibirsk region
L-421	nutans	ICG-8243/K-27058	Russia, Novosibirsk region
Manych 459	nutans	ICG-8266/K-29345	Russia, Rostov region
Mayak	nutans	ICG-8159/K-29622	Russia, Krasnoyarsk region
Medikum	medicum	ICG-8322/K-29002	Kazakhstan
Melius	nutans	ICG-8529	Switzerland
Merit 57	nutans	ICG-8530	USA

Mestny Dagestanian	nigrum nudum	ICG-8459/K-15010	Russia, Primorsky Krai
Mestny Ethiopian	nigrum nudum	ICG-8461/K-20042	Russia, Dagestan
Mestny Primorsky	pallidum	ICG-8211/K-15117	Russia, Primorsky Krai
Mestny Yakutian	nigrum nudum	ICG-8203/K-7979	Russia, Yakutia
Moskovsky 121	nutans	ICG-8236/K-19417	Russia, Moscow region
Mutant 68	nudum	ICG-8055/K-28163	Russia, Novosibirsk region
Narymchanin	ricotense	ICG-8287/K-27039	Russia, Tomsk region
NGB 122412	nutans	ICG-8531	Russia, Novosibirsk region
Nikita	nutans	ICG-8075/K-30900	Russia, Kemerovo region
Nosovsky 11	nutans	ICG-8255/K-29465	Ukraine
Novosibirsky 80	nutans	ICG-8437/K-26848	Russia, Novosibirsk region
Nutans 970	nutans	ICG-8040/K-21794	Kazakhstan
Obskoy	nutans	ICG-8031/K-24709	Russia, Novosibirsk region
Omsky 13709	nutans	ICG-8242/K-17843	Russia, Omsk region
Omsky 85	pallidum	ICG-8042/K-27927	Russia, Omsk region
Omsky golozyorny 1	nudum	ICG-8457/K-30919	Russia, Omsk region
Omsky golozyorny 2	coeleste	ICG-8458/-	Russia, Omsk region
Orenburgsky kormovoy	nutans	ICG-8248/K-29496	Russia, Orenburg region
Oskar	nutans	ICG-8463/K-31040	Russia, Krasnoyarsk region
Pallidum 394	pallidum	ICG-8174/K-20916	Russia, Buryatia
Prikumsky 14	nutans	ICG-8286/K-25075	Russia, Stavropol Territory
Reyd	nutans	ICG-8014/K-28885	Russia, Sverdlovsk region
Sasha	medicum	ICG-8532/K-31110	Russia, Omsk region
Selection from Tyal	coeleste	ICG-8226/-	Russia, Novosibirsk region
Severny	pallidum	ICG-8066/K-25298	Russia, Leningrad region
Signal	nutans	ICG-8032/K-30846	Russia, Altai region
Simvol	nutans	ICG-8077/K-27502	Russia, Rostov region
Slavyansky	nutans	ICG-8139/K-29611	Russia, Voronezh region
Sobolyok	ricotense	ICG-8375/K-30245	Russia, Krasnoyarsk region
Svetik	parallelum	ICG-8038/K-29009	Russia, Arkhangelsk region
Symbat	nutans	ICG-8533	Kazakhstan
Taganay	nutans	ICG-8029/K-29001	Russia, Chelyabinsk region
Talan	nutans	ICG-8534	Russia, Novosibirsk region
Tanay	nutans	ICG-8535	Russia, Novosibirsk region
Tarsky 1	nutans	ICG-8214/K-30150	Russia, Omsk region
Tatum	nutans	ICG-8536	Germany
Temp	nutans	ICG-8279/K-22055	Russia, Krasnodar region
Vikont	nutans	ICG-8520/K-30301	Russia, Krasnodar region
Viner	nutans	ICG-8136/K-8514	Russia, Kirov region
Viner mutant	nutans	ICG-8293/-	Russia, Leningrad region
Vorsinsky 2	nutans	ICG-8521	Russia, Altai region
Vybor	ricotence	ICG-8130/K-29409	Russia, Moscow region
Wial	horsfordianum	ICG-8392/-	USA
Zalarinets	nutans	ICG-8209/K-16955	Russia, Irkutsk region
Zernogradsky 86	nutans	ICG-8065/K-25936	Russia, Rostov region
Zolotnik	medicum	ICG-8439/K-30845	Russia, Altai region
